# Preparation of GO/Diatomite/Polyacrylonitrile Functional Separator and Its Application in Li–S Batteries

**DOI:** 10.3390/ma17040789

**Published:** 2024-02-06

**Authors:** Jing Yang, Wenjie Xiao, Xiaoyu Wu, Yitao Zha, Sainan Liu

**Affiliations:** 1 School of Minerals Processing and Bioengineering, Central South University, Changsha 410083, China; 215612104@csu.edu.cn (J.Y.); 225612081@csu.edu.cn (W.X.); 235611138@csu.edu.cn (X.W.); 2 School of Materials Science and Engineering, Central South University, Changsha 410083, China; 233111018@csu.edu.cn

**Keywords:** Li–S batteries, LPS, shuttle effect, GO/DE/PAN functional separator

## Abstract

Lithium–sulfur (Li–S) batteries have received extensive attention due to their numerous advantages, including a high theoretical specific capacity, high energy density, abundant reserves of sulfur in cathode materials, and low cost. Li–S batteries also face several challenges, such as the insulating properties of sulfur, volume expansion during charging and discharging processes, polysulfide shuttling, and lithium dendritic crystal growth. In this study, a composite of a porous multi-site diatomite-loaded graphene oxide material and a PAN fiber membrane is developed to obtain a porous and high-temperature-resistant GO/diatomite/polyacrylonitrile functional separator (GO/DE/PAN) to improve the electrochemical performance of Li–S batteries. The results show that the use of GO/DE/PAN helps to inhibit lithium phosphorus sulfide (LPS) shuttling and improve the electrolyte wetting of the separator as well as the thermal stability of the battery. The initial discharge capacity of the battery using GO/DE/PAN is up to 964.7 mAh g^−1^ at 0.2 C, and after 100 cycles, the reversible capacity is 683 mAh g^−1^ with a coulombic efficiency of 98.8%. The improved electrochemical performance may be attributed to the porous structure of diatomite and the layered composite of graphene oxide, which can combine physical adsorption and spatial site resistance as well as chemical repulsion to inhibit the shuttle effect of LPS. The results show that GO/DE/PAN has great potential for application in Li–S batteries to improve their electrochemical performance.

## 1. Introduction

With the gradual increase in the demand for energy, it is becoming increasingly apparent that using fossil fuels to meet this demand is problematic [1,2,3]. Moreover, with natural renewable energy, such as solar, wind, and water energy, being intermittent and unstable in their use, it is difficult to achieve a large-scale stable renewable energy supply; thus, the development of energy storage equipment is particularly important [4,5]. The purpose of rechargeable secondary battery systems is to convert stored chemical energy into electrical energy and exhibit the characteristics of environmental friendliness, high energy density, a high-efficiency conversion rate, and safety [6,7]. Theoretically, Li–S batteries have a high specific capacity (~1675 mAh/g, about 10 times that of traditional lithium-ion batteries) and high energy density (~2600 Wh/kg, about 6 times that of traditional lithium-ion batteries) [8]. However, the development and application of Li–S batteries are limited by their poor thermal stability, volume expansion during charging and discharging, LPS shuttling, lithium dendrite growth, etc. [9,10]. In particular, the thermal stability and diffusion of LPS determine the safety and actual specific capacity as well as the cycle life of the battery; therefore, improving the thermal stability of batteries and inhibiting LPS shuttling in the electrolyte are key to realizing the application of Li–S batteries [11].

For the key problems of Li–S batteries, various strategies have been introduced, such as the incorporation of carbon conducting materials, interface modulation, electrode surface and composition design, etc. [12,13]. Moreover, the introduction of functional sandwiches or multifunctional separators has become a research focal point [14,15,16]. Separators have a rich pore structure that can be infiltrated by the electrolyte and allow Li^+^ migration; however, the pore structure of separators also provides a channel for polysulfide ion transfer [17]. Functionalized separators improve thermal stability while inhibiting LPS shuttling through physical and chemical adsorption, thus enhancing the electrochemical performance of the battery [18,19]. At present, carbon materials, polymers, metals, and metal compounds are widely used in Li–S battery separator modification and functional sandwich designs intended to solve the problem of the shuttle effect [20,21]. Multifunctional separators that have been used in Li–S batteries include the following: carbon-modified [22,23], polymer-modified [24,25,26], oxide-modified [27,28,29], and metal-sulfide-modified separators [30,31]. Compared with other modification methods, carbon materials have a large specific surface area and good electrical conductivity, can adsorb polysulfides to inhibit the shuttle effect, and can also play the role of the upper collector to promote rapid electron transport and enhance the utilization of active substances [32,33,34].

Here, GO/DE/PAN based on diatomite mineral materials is designed as a Li–S battery separator using electrostatic spinning technology and by performing electrochemical tests. Diatomite has unique intrinsic properties such as a rich natural pore structure and negative charge groups as well as Si-OH-rich groups and active adsorption sites on the surface [35,36], which can be used as adsorption sites to adsorb and immobilize LPS, thus slowing down the shuttling phenomenon of LPS. GO/DE composite materials also exhibit the characteristics of oxygen-containing functional groups, which have outstanding sulfur adsorption. GO/DE/PAN can combine physicochemical adsorption with spatial site resistance and charge repulsion to inhibit the LPS shuttle effect, and the wettability of the electrostatic spinning film is also salient.

## 2. Materials and Methods

### 2.1. Purification of Diatomite

The treatment process of water washing-acid leaching-water washing was used to decontaminate second-grade diatomite from Changbai Mountain. Firstly, 20 g of raw diatomite was dissolved in 1500 mL of pure water, stirred for 12 h, and then left to stand for 12 h. The lower layer of precipitate was taken, dried, and passed through a 400-mesh sieve; then, 5 g of diatomite was dissolved in 2 mol/L of diluted sulphonic acid in a ratio of 1:20, stirred for 3 h, filtered, and dried. Afterward, the acid-washed diatomite was dissolved in 1000 mL of pure water, stirred for 12 h, and then left to stand for 12 h. Finally, the lower layer of precipitate was taken.

### 2.2. Preparation of GO/DE Composite Material

Firstly, graphene oxide was dispersed as 0.8 mg mL^−1^ of GO suspension, and 0.2 g of diatomite was dispersed in 200 mL of GO suspension. The resulting mixture was subjected to microwave branching treatment using a UV microwave ultrasonic synthesis device, with a microwave time of 120 min and a stirring heating temperature of 50 °C. Finally, the black powder was centrifuged and freeze-dried to obtain a GO/DE composite material.

### 2.3. Preparation of GO/DE/PAN Functional Separator

Solutions of 7 mL of dimethylformamide and 3 mL of acetone were mixed evenly to obtain a solvent at 20~25 °C; then, 1 g of PAN, 0.075 g of GO/DE composite material, and 0.015 g of PVDF were added to the solution while stirring. The materials were completely dissolved and dispersed using magnetic stirring at 700 rpm for 12 h at room temperature and then ultrasonicated at 30 °C for 30 min to make the dispersion homogeneous. The resulting dispersion was left to defoam for 2 h to obtain the GO/DE/PAN dispersion. Subsequently, 10 mL of GO/DE/PAN dispersion was extracted with a medical syringe, a 22-gauge flat-mouth stainless steel needle was used to spin on the electrospinning device, and the nanofibers obtained from spinning were collected on aluminum foil. The distance between the needle and the receiving plate was 22 cm, the spinning voltage was 20 KV, the ambient temperature was 25 °C, the humidity was 30%, and the feeding speed was 0.8 mL/h. The nanofiber membrane was vacuum-dried at 70 °C for 12 h and hot-pressed into a 20~30 μm nanofiber base film, which was GO/DE/PAN.

### 2.4. Synthesis of S/C Cathode Materials

S and acetylene black (ACET) were mixed at a mass ratio of 7:3, and the material was transferred to an autoclave reactor in a glove box and heated at 155 °C for 12 h after full grinding. Subsequently, a sulfur–carbon composite material was obtained after cooling and full grinding. The cathode material, PVDF (binder), and Super P (conductive agent) were mixed at a mass ratio of 8:1:1, and an appropriate amount of NMP was added dropwise to the mixed material and stirred at a constant speed in a magnetic stirrer for 6 h to form a homogeneous slurry. The homogenizing slurry was scraped onto commercial aluminum foil at a thickness of 150 μm and then vacuum-dried at 60 °C for 12 h. Finally, the sulfur cathode was stamped into small discs with a diameter of 14 mm, the complete positive discs were weighed on an electronic balance with high precision, and the amount of active material was calculated according to the proportion.

### 2.5. Material Characterization

The crystal structure of the composites was measured using X-ray diffraction (XRD, Rigaku DX-2500, Rigaku, Tokyo, Japan), which was obtained using Cu Kα radiation (λ = 1.5418 Å) in the range of 2θ from 5° to 80°. Fourier transform infrared spectra (FTIR) were recorded using a Shimadzu FTIR 8120 spectrometer (Shimadzu, Kyoto, Japan) in the range of 400 to 4000 cm^−1^. The total specific surface area of the materials was analyzed using a 4-station fully automated specific surface area analyzer, model APSP 2460, Micromeritics, Norcross, GA, USA. Samples were tested for nitrogen adsorption and desorption under 77 k liquid nitrogen conditions to analyze the total specific surface area of the material. The morphology and structure were characterized using a field emission scanning electron microscope (SEM, FEI Nova NanoSEM230, 20 kV). A JY-82 C video contact angle meter was used to determine the water contact angle.

### 2.6. Electrochemical Measurements

GO/DE/PAN was placed between the cathode and the lithium tabs, and the CR2032-type coin batteries were assembled in an Ar-filled glove box. An original Celgard2400 (PP) was used as a comparison. Cyclic voltammetry (CV) was performed over a voltage range of 1.7–2.8 V at a scan rate of 0.1 mV s^−1^ on a CHI611C constant potential meter (Shanghai Chenhua, Shanghai, China). Electrochemical impedance spectroscopy (EIS) was performed using a CHI611C constant potential meter in a frequency range of 100 kHz–0.01 Hz. Constant current discharge/charge performance of the batteries was evaluated at 1.7–2.8 V using a CT2001A battery test instrument (LAND Electronic Co., Wuhan, China).

## 3. Results and Discussion

**Figure 1:**
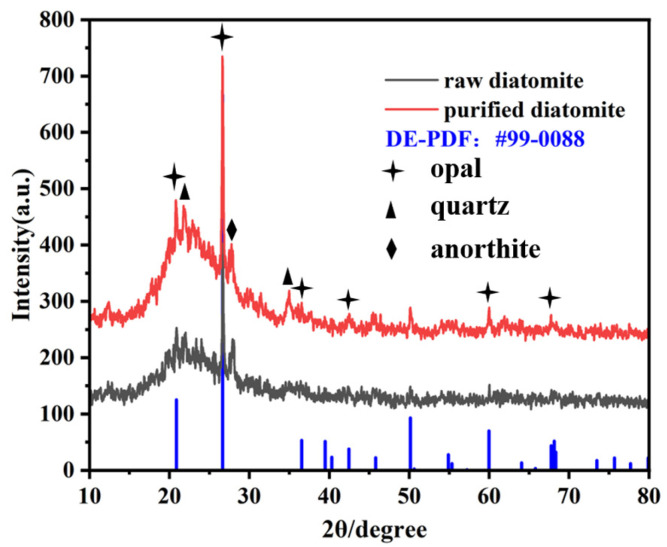
XRD patterns of raw and purified diatomite.

An XRD characterization of raw diatomite and experimental diatomite after purification is shown in Figure 1. X-ray diffraction was used to measure the crystal structure of the composites; the main components of diatomite are amorphous SiO_2_ (accounting for up to 80–94%), Al_2_O_3_ (3–6%), and Fe_2_O_3_ (1–1.5%) [37], as well as a small number of other oxides and organic matter, etc. The mineral composition of diatomite is mainly opal, and diffraction peaks of quartz and dolomite also appeared in the sample, which are impurities in diatomite. The following figure shows that the characteristic peaks of diatomite are clearly enhanced after purification.

**Figure 2:**
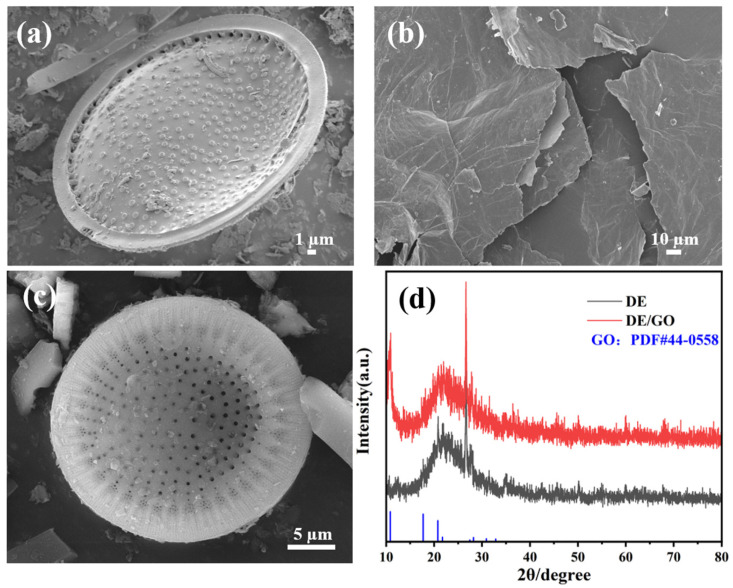
SEM images of (**a**) DE, (**b**) GO; (**c**) GO/DE. (**d**) XRD photographs of DE and GO/DE composite material.

As shown in Figure 2a, the algal discs of diatomite are characterized by a porous morphology with highly developed macro- and mesoporous structures with surface pore diameters ranging from 30 to 55 μm. Figure 2b shows the surface scanning of graphene oxide, which can be observed to be in the form of phosphorus flakes. The morphology of the GO/DE composites is shown in Figure 2c. The surface of graphene-oxide-loaded diatomite is covered by translucent folded flakes, which is a typical morphology feature of GO nanosheets [38] and mainly concentrates around the algal discs and beside the individual diatomite fragments in a cascade fashion. As presented in Figure 2d, the crystal structures of diatomite and graphene oxide/diatomite composites were characterized using X-ray diffraction (XRD). The strong peaks at 2θ = 21.8° and 26.8° are the 100-crystal plane and 101-crystal plane diffraction peaks of diatomite, respectively, and the strong peak at 2θ = 11.6° is the 001-crystal plane diffraction peak of graphene oxide. This indicates that diatomite and graphene oxide coexist in the sample.

**Figure 3:**
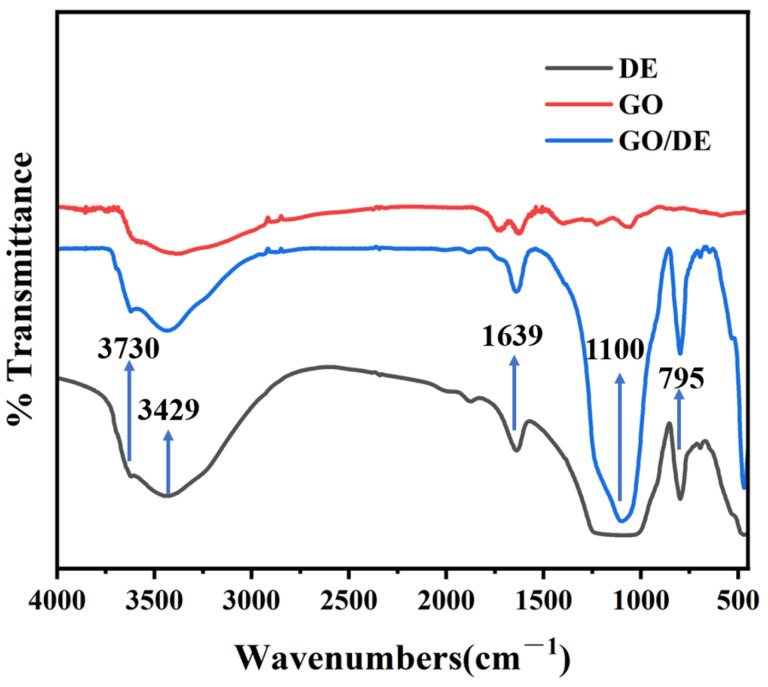
FTIR spectra of DE, GO, and GO/DE composite materials.

Figure 3 shows the Fourier transform infrared (FTIR) spectra of the DE, GO, and GO/DE composite materials. Diatomite has relatively broad Si-OH stretching vibration peaks near 3700–3050 cm^−1^, indicating that there are a large number of silicon hydroxyl groups on the surface of diatomite. The weak peak at 3730 cm^−1^ is due to the O-H stretching of diatomite hydroxyl groups, and the absorption peaks at 1639 cm^−1^ are attributed to aqueous hydroxyl (-OH). The absorption peaks at 1100 cm^−1^, 795 cm^−1^, and 468 cm^−1^ are the symmetric contraction vibration peaks and bending vibration peaks in the transverse and longitudinal directions of the Si-O-Si bond, respectively. There are several characteristic absorption peaks in the FTIR pattern of GO: the broad absorption peak at the wavelength of 3430 cm^−1^ corresponds to the O-H stretching vibration, the characteristic absorption peak at 1640 cm^−1^ corresponds to the stretching vibration of the C=C bond on the sp^2^ carbon skeleton structure, the corresponding absorption peak at 1098 cm^−1^ is the C-OH bending vibration, and 1186 cm^−1^ is the C-O-C stretching vibration. The large quantities on the surface of diatomite Si-OH (silicone hydroxyl) groups can provide active sites to graft graphene oxide, and the absorption peaks of the FTIR spectra of GO/DE composites are basically the same as those of the FTIR spectra of DE. The peak at 3445.53 cm^−1^ in the FTIR spectrum of GO/DE composites is formed by free silanol groups (SiO-H) at the surface, which are the main components of the composites, and the absorption peaks located at 1640.56 cm^−1^, 1100 cm^−1^, 800.36 cm^−1^, and 467 cm^−1^ are the (H-O-H) bending vibration of water in diatomite, the asymmetric stretching of the Si-O-Si bond, the Si-O stretching of the silanol group, and the Si-O-Si bending vibration, respectively.

**Figure 4:**
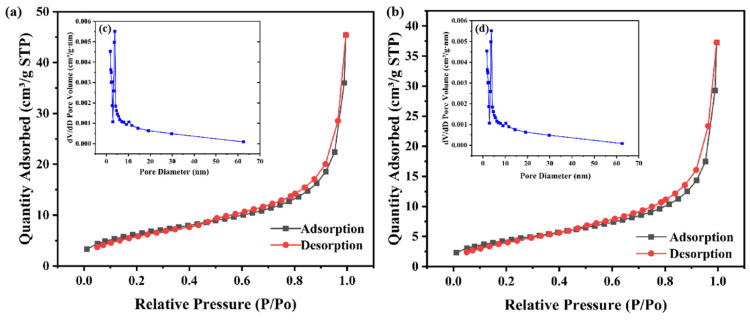
(**a**,**b**) N_2_ adsorption/desorption isotherms, (**c**,**d**) pore size distribution of DE and GO/DE.

The materials were tested using an N_2_ adsorption–desorption isotherm to accurately measure the specific surface area of the material before and after grafting. As presented in Figure 4, the adsorption–desorption curves of diatomite and graphene oxide/diatomite composites are typical type II adsorption isothermal curves, and the curves of the two are similar. The curves indicate that both monolayer and multilayer adsorption occur, and the pore size of each material is different, exhibiting mainly mesoporous pores and concentrated at 5–10 nm. The abundant mesoporous structure can not only inhibit the shuttle of polysulfides through physical barriers but also provide a transport channel for Li^+^, as well as store more electrolytes to shorten the ion transport distance, which is conducive to the progress of charge–discharge reactions. The data show that the specific surface area of diatomite is 25.8118 m^2^/g, and the specific surface area of the composite material is 23.4909 m^2^/g after grafting graphene oxide on diatomite. The specific surface area does not change greatly, indicating that the pores of the material are mainly provided by diatomite, and the introduction of graphene oxide does not cover or block the pores.

**Figure 5:**
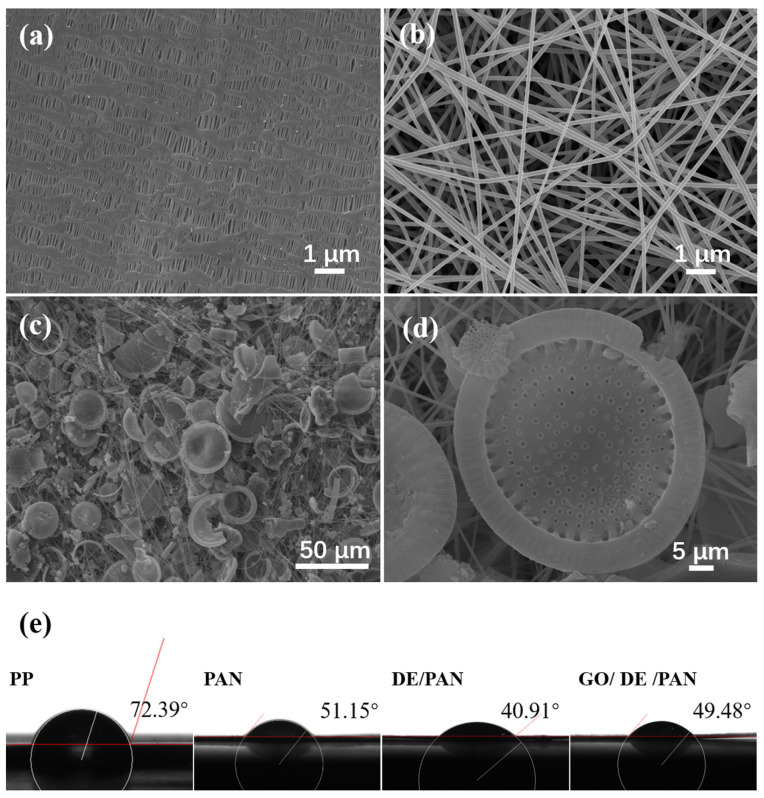
SEM images of (**a**) PP, (**b**) PAN, (**c**) DE/PAN, and (**d**) GO/DE/PAN; (**e**) separator contact angles of PP, PAN, DE/PAN, and GO/DE/PAN.

The surface and internal structure of commercial PP separators, blank PAN separators, and GO/DE/PAN were studied using scanning electron microscopy (SEM). As shown in Figure 5a, the surface of the commercial PP separator is flat, and the inside is a staggered network structure, which is densely interwoven, and the wire mesh is thicker. The internal nanofibers of the PAN separator (Figure 5b) are staggered to form pores of different sizes, with fiber diameters ranging from 30 to 50 nm, and the internal void structure of the separator can be adjusted according to the pressure of electrospinning and the concentration of the spinning solution, such that the staggered pore density can effectively ensure the rapid transfer of ions and realize the diffusion of polysulfides to the anode. As can be seen from the electron microscope scan of GO/DE/PAN (Figure 5c), the GO/DE composite material is dispersed between nanofibers, and the nanofiber filaments form a wrapping around the outer surface, which introduces a finer void compared to the original blank nanofiber membrane. Combined with the Si-OH-rich groups and negative groups of the GO/DE composite material, this can significantly inhibit the LPS shuttle effect during the reaction of Li–S batteries.

In addition, the wetting ability of different separators was tested using contact angle measurements. As shown in Figure 5e, the contact angle of the commercial PP separator is 72.39°, exhibiting poor wettability to the electrolyte. The contact angle of the blank PAN separator is 51.15°, which is lower than that of the widely used PP separator. The contact angle of the diatomite/polyacrylonitrile separator (DE/PAN) is 40.91°, which is again lower than that of the blank PAN separator; for GO/DE/PAN, the contact angle is 49.48°. With the addition of graphene oxide, the wettability of the separator is reduced; however, it is still higher than that of PP and PAN, indicating that the composite separator exhibits outstanding wettability performance, potentially ensuring the full absorption and wetting of the electrolyte, accelerating ion transfer efficiency, and contributing to the improvement in the electrochemical performance of the battery.

**Figure 6:**
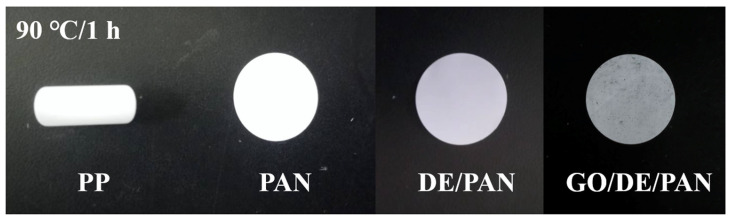
Images of the PP, PAN, DE/PAN, and GO/DE/PAN separators after heating at 90 °C for 1 h.

Figure 6 shows the surface morphology of PP, PAN, DE/PAN, and GO/DE/PAN after being heated at 100 °C for 1 h. It can be observed that the PP separator shrinks significantly after being heated, with a shrinkage rate of up to 74%. In contrast, PAN, DE/PAN, and GO/DE/PAN did not shrink, indicating that the thermal stability of the GO/DE/PAN separator, obtained using electrostatic spinning technology, was greatly improved and that the use of GO/DE/PAN in Li–S batteries can ensure that the internal chemical reactions of the batteries can be carried out normally at high temperatures, which can help to improve the thermal stability and electrochemical performance of the batteries.

**Figure 7:**
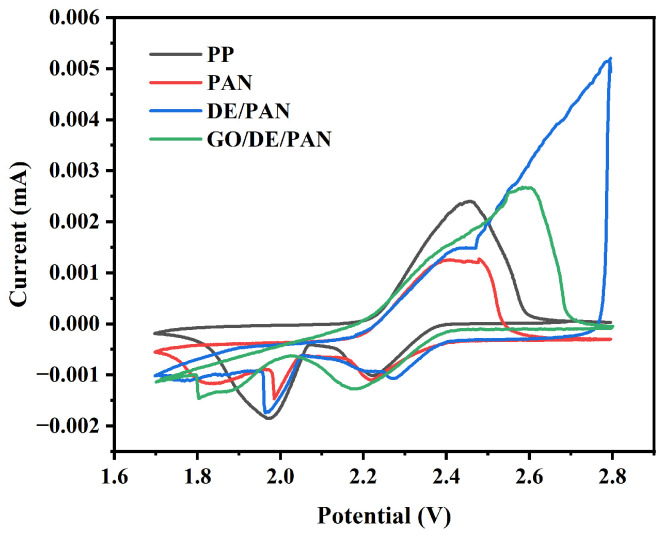
CV curves of PP, PAN, DE/PAN, and GO/DE/PAN separators.

Cyclic voltammetry (CV) tests were performed on the lithium–sulfur batteries assembled with PP, PAN, DE/PAN, and GO/DE/PAN, with a voltage window of 1.7–2.8 V and a scanning rate of 0.1 mV/s. The CV curves are shown in Figure 7. From the curves, two typical reduction peaks can be observed: the reduction peak near 2.3 V corresponds to the conversion of S_8_ to soluble long-chain lithium polysulfide, and the reduction peak near 2.1 V corresponds to the further conversion of long-chain lithium polysulfide to insoluble Li_2_S/Li_2_S_2_. The oxidation peak around 2.3 V corresponds to the reverse oxidation of Li_2_S/Li_2_S_2_ to elemental sulfur. As can be seen from the figure, the oxidation peak of the GO/DE/PAN shifted to a lower potential, indicating that the battery assembled with GO/DE/PAN may promote the oxidation reaction. Moreover, the reduction peak of the battery with the GO/DE/PAN assembly has a higher peak current and a larger peak area near 2.3 V, indicating that the accelerated migration of Li^+^ and the rapid conversion of lithium polysulfide contribute to the high capacity.

**Figure 8:**
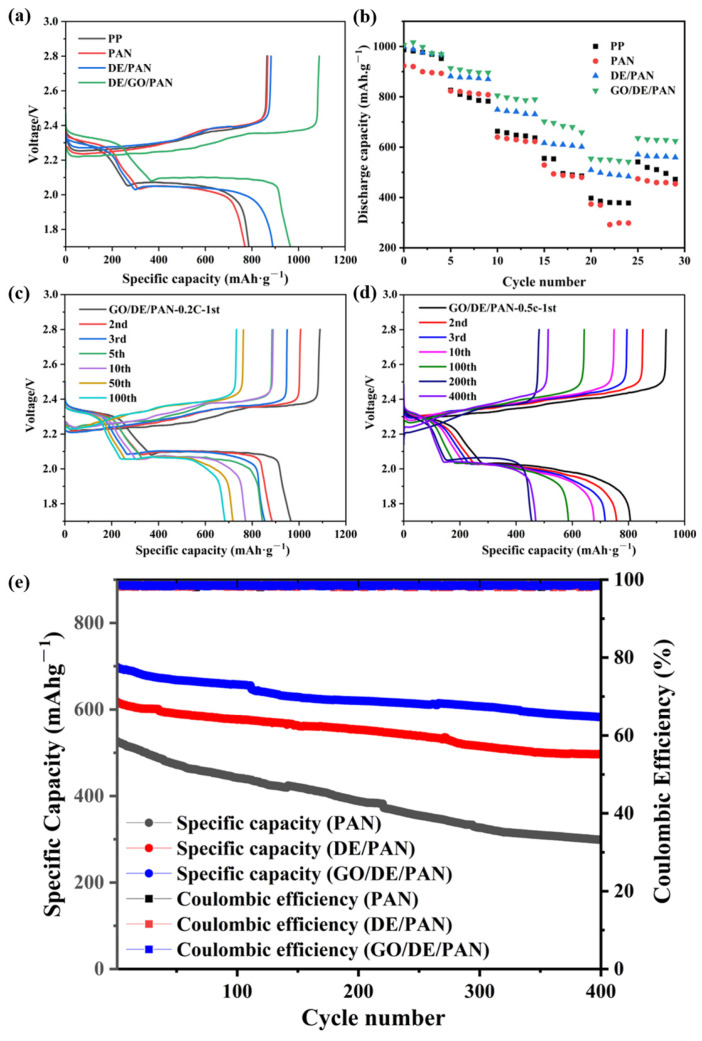
(**a**) Discharge–charge voltage profiles and (**b**) rate performance of the LSBs with PP, PAN, DE/PAN, and GO/DE/PAN; discharge–charge voltage profiles of the LSBs with GO/DE/PAN at a current density of (**c**) 0.2 C and (**d**) 0.5 C; (**e**) cycling performance of the LSBs with PAN, DE/PAN, and GO/DE/PAN.

As shown in Figure 8a, the batteries with different separators were tested for charging and discharging and compared with the PP separators. The initial discharge-specific capacities of PP, PAN, DE/PAN, and GO/DE/PAN are 786.5, 768.8, 888.7, and 964.7 mAh g^−1^, respectively, at 0.2 C. Compared with the PP separator, the initial discharge-specific capacity of blank PAN is relatively low. This indicates that, during application, the electrochemical performance of the blank PAN is not as good as that of the PP separator, although the structures of the two separators are similar. The initial discharge-specific capacities of both DE/PAN and GO/DE/PAN are greatly improved compared to blank PAN and are higher than that of PP. This is due to the introduction of more mesoporous diatomite materials with different pores in both DE/PAN and GO/DE/PAN compared to blank PAN, which inhibits polysulfide shuttling to a certain extent, as well as the high conductivity of graphene oxide in GO/DE/PAN, which can also effectively shorten the transmission channels of electrons and ions. At the same time, both have better wettability to the electrolyte, which provides more media for Li^+^ migration and increases the Li^+^ migration rate. The multiplication capacity of batteries using different separators has also been investigated. As shown in Figure 8b, the battery with PP performs poorly, providing 987, 831.7, 664, 558, and 399 mAh g^−1^ at 0.1, 0.2, 0.5, and 1 C, respectively, demonstrating 543.1 mAh g^−1^ when returning to 0.5 C. In contrast, the batteries with PAN, DE/PAN, and GO/DE/PAN provide initial discharge capacities of 924, 997, and 1018 mAh g^−1^ at 0.1 C. GO/DE/PAN and PAN provide the highest and lowest initial discharge capacities, respectively; however, the DE/PAN and GO/DE/PAN separators both provide higher initial discharge capacities than the battery with PP. In addition, the batteries assembled with GO/DE/PAN provide high reversible capacities of 1017, 914, 808, 707, and 560 mAh g^−1^ at 0.1 C, 0.2 C, 0.5 C, and 1 C, respectively, demonstrating a high capacity of 637 mAh g^−1^ when returning to 0.5 C. The introduction of GO/DE composites is beneficial for the storage capacity of liquid electrolytes, increases the transport speed of ions/electrons, and, consequently, leads to a significant enhancement of the electrochemical performance of Li–S batteries.

Figure 8c shows the charge–discharge curves of Li–S batteries with GO/DE/PAN at 0.2 C for different cycles, and two discharge plateaus can be observed, which is consistent with the results of the two reduction peaks in the CV curve. The GO/DE/PAN separator can still provide a discharge-specific capacity of 683 mAh g^−1^ after 100 cycles at 0.2 C, and with a clear discharge platform after 100 cycles, thus confirming its good electrochemical performance and excellent cycling stability. Figure 8d shows the charge–discharge curves of the GO/DE/PAN separator at 0.5 C with different cycles. The battery with the GO/DE/PAN separator can provide a discharge-specific capacity of 808 mAh g^−1^ in the first revolution at 0.5 C and continues to provide a discharge-specific capacity of 467 mAh g^−1^ after 400 cycles, demonstrating that the battery with GO/DE/PAN ensures good cycling performance even at this current density. Figure 8e further illustrates the cycling performance of batteries with GO/DE/PAN and others for 400 cycles at a current density of 1 C. The initial discharge capacity of the blank PAN is only 526.7 mAh g^−1^, and the capacity decreases sharply with an increase in the number of cycles. The initial discharge capacity of DE/PAN is relatively high, at 617.8 mAh g^−1^, and its discharge capacity decreases slowly with a further increase in the number of cycles, which means that the introduction of diatomite can maintain the stability of the battery to a certain degree. The initial discharge capacity of the battery with GO/DE/PAN reaches 698.1 mAh g^−1^, the reversible capacity of 657.8 mAh g^−1^ is maintained after 100 cycles, and the capacity attenuation rate is only 0.05%, indicating that the battery has good cycling stability.

**Figure 9:**
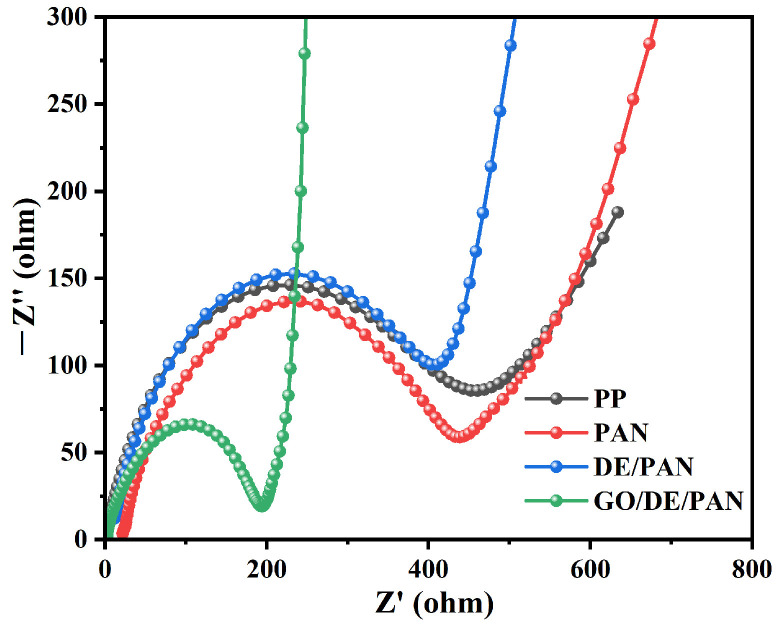
EIS spectra of PP, PAN, DE/PAN, and GO/DE/PAN separators.

Figure 9 shows the Nyquist plot of the electrochemical impedances of GO/DE/PAN, PP, PAN, and DE/PAN, and the intersection of the Nyquist plot with the horizontal axis is the body resistance of the separator. Compared with PP, PAN, and DE/PAN, the Rct value of the battery with GO/DE/PAN demonstrates a significant reduction, indicating that GO/DE/PAN enhances the electrochemical reaction kinetics, thus facilitating the fast transport of Li^+^ and the efficient blocking effect of LPS. This is also consistent with the excellent electrochemical performance of batteries with GO/DE/PAN.

Table 1 shows the performance of batteries with GO/DE/PAN and others, further illustrating the application advantages of GO/DE/PAN in Li–S batteries.

## 4. Conclusions

In summary, the GO/DE/PAN separator, prepared using electrostatic spinning technology, greatly enhances the electrochemical performance of Li–S batteries. GO/DE/PAN, as a kind of high-efficiency ionic sieve with a porous multi-site diatomite-loaded graphene oxide material, can repel, hinder, and reduce the transfer of polysulfide anion; effectively reduce the shuttle effect; and improve the shuttle efficiency of Li^+^. In addition, the utilization of GO/DE/PAN further improves sulfur utilization. The lower thermal shrinkage and contact angle also ensure the thermal stability and efficiency of the battery. These significant advantages are key to improving the specific capacity and cycling stability of Li–S batteries. The battery with GO/DE/PAN demonstrates an initial discharge capacity of 1018 mAh g^−1^ at 0.1 C and maintains an initial discharge capacity of 698.1 mAh g^−1^ even at a high current density of 1 C. The discharge capacity is 683 mAh g^−1^ with a Coulombic efficiency of 98.9% after 100 cycles at a current density of 0.2 C. Therefore, introducing GO/DE composites into PAN nanofiber separators to prepare GO/DE/PAN and employing it as a Li–S battery separator is a potentially effective strategy to suppress the shuttle effect and enhance the electrochemical performance of Li–S batteries.

## Figures and Tables

**Table 1:** Summary of Li–S performance with different separators.

	Specific Charge	C Rate		
Membrane	(mAh g^−1^)	(C)	Cycles	Ref.
PP/PAA	562	0.5	600	[39]
PP/Nafion/Cu-MOF	680	0.5	300	[40]
PP/PDA/g-CN	764	0.5	500	[41]
PP/Naon/super P	807	0.5	250	[42]
PP/silicone/PDA	982	1	1000	[43]
PP/Co-carbon/PDDA	872	2	1200	[44]
PP/PSS	1300	0.05	30	[45]
PP/PANI/CFP	723	1	100	[46]
MnS/CNF	714	0.5	200	[47]
PAN@APP	634	1	400	[48]
N-CNFs	986	0.2	200	[49]
S-CNTs/CoNCNFs/PVDF	933	0.2	400	[50]
GO/DE/PAN	964.7	0.2	100	this paper

## Data Availability

Data are contained within the article. The data presented in this study are available on request from the corresponding author.
